# Long non-coding RNAs in *Sus scrofa* ileum under starvation stress

**DOI:** 10.5713/ab.21.0483

**Published:** 2022-03-02

**Authors:** Shu Wang, Yi Jia Ma, Yong Shi Li, Xu Sheng Ge, Chang Lu, Chun Bo Cai, Yang Yang, Yan Zhao, Guo Ming Liang, Xiao Hong Guo, Guo Qing Cao, Bu Gao Li, Peng Fei Gao

**Affiliations:** 1 Department of Animal Sciences, Shanxi Agricultural University, Taigu, Shanxi Province 030801, China; 2 Inner Mongolia Mengniu Dairy(GROUP) CO., LTD, Shengle Economic Zone, Helingeer Hohhot, Inner Mongolia 011500, China

**Keywords:** Co-expression Network, LncRNA, Pigs, Starvation Stress

## Abstract

**Objective:**

In this study, we aimed to identify long non-coding RNAs (lncRNAs) that play important roles in starvation stress, analyze their functions, and discover potential molecular targets to alleviate starvation stress to provide a theoretical reference for subsequent in-depth research.

**Methods:**

We generated a piglet starvation stress animal model. Nine Yorkshire weaned piglets were randomly divided into a long-term starvation stress group (starved for 72 h), short-term starvation stress group (starved for 48 h), and the control group. LncRNA libraries were constructed using high-throughput sequencing of piglet ileums.

**Results:**

We obtained 11,792 lncRNAs, among which, 2,500 lncRNAs were novel. In total, 509 differentially expressed (DE)lncRNAs were identified in this study. Target genes of DElncRNAs were predicted via cis and trans interactions, and functional and pathway analyses were performed. Gene ontology functions and Kyoto encyclopedia of genes and genomes analysis revealed that lncRNA-targeted genes mainly participated in metabolic pathways, cellular processes, immune system processes, digestive systems, and transport activities. To reveal the mechanism underlying starvation stress, the interaction network between lncRNAs and their targets was constructed based on 26 DElncRNAs and 72 DEmRNAs. We performed an interaction network analysis of 121 DElncRNA–DEmRNA pairs with a Pearson correlation coefficient greater than 0.99.

**Conclusion:**

We found that MSTRG.19894.13, MSTRG.16726.3, and MSTRG.12176.1 might play important roles in starvation stress. This study not only generated a library of enriched lncRNAs in piglets, but its outcomes also provide a strong foundation to screen key lncRNAs involved in starvation stress and a reference for subsequent in-depth research.

## INTRODUCTION

The World Health Organization (WHO) reports that since 1975, the number of obese people in the world has grown nearly three-fold, and currently, nearly 20 million adults are overweight or obese [1]. Obesity not only affects one’s physical appearance but also causes various diseases [2] that burden the economy and the medical and health infrastructure. At present, popular methods of weight loss include dieting, targeted drug therapy, and surgical liposuction. Weight loss based on long-term fasting has significant effects, but it is not healthy [3]. Short-term starvation stress might be beneficial for cardiometabolic health by decreasing insulin resistance, blood pressure, and oxidative stress [4]. Long-term starvation stress can lead to a deficit of effective nutrients, increase levels of corticosterone, the stress hormone, and inhibit cardiovascular function [5].

The intestine is the main site of the digestion, absorption, and metabolism of nutrients in the body. The host intestinal mucosa directly or indirectly participates in metabolism, and its function is closely related to metabolic diseases such as obesity, diabetes, and allergies; moreover, it plays an active role in immune regulation [6]. Studies have reported that starvation can cause changes in the intestinal structure, damage the intestinal barrier, decrease the villus height-to-crypt depth (V/C) ratio, and dysregulate the intestinal flora, which eventually leads to metabolic disorders [7].

Long non-coding RNAs (lncRNAs) are a class of non-coding RNAs longer than 200 nucleotides that are widely present in the nucleus and cytoplasm [8]. LncRNAs participate in the regulation of various biological processes (BPs), such as gene expression, transcriptional activation, cell cycle regulation, and disease occurrence [9]. Recent studies have surmised that an increasing number of lncRNAs play important roles in maintaining intestinal epithelial homeostasis, promoting the proliferation and apoptosis of intestinal epithelial cells, and regulating the intestinal barrier [10–12]. For example, lncRNA H19 promotes the expression of miR-675-5p and miR-675-3p, which downregulate the levels of tight junction proteins 1 (TJP1, also called ZO-1) and E-cadherin, destroying the intestinal epithelial barrier [10]. In mice with starvation-induced mucosal atrophy, lncRNA uc.173 promotes the proliferation of intestinal epithelial cells by reducing the expression of miR-195 [11]. Transcriptome sequencing performed on ulcerative colitis tissue revealed lncRNA BC012900 overexpression. Furthermore, the overexpression of this lncRNA promotes intestinal epithelial cell apoptosis [12].

Owing to the availability of genetic information, easy genetic modification, and low cost of rearing, mice are the preferred *in vivo* models for studying human diseases [5,13]. However, significant differences exist in metabolic and physiological characteristics between humans and mice, which prevents researchers from applying findings from murine studies to humans for metabolism-related disease prevention and intervention strategies [14]. In contrast, pigs are very similar to humans in terms of metabolic characteristics and organ development; therefore, pigs are an ideal animal model for human energy metabolism research [15]. Thus, in this study, we subjected weaned piglets to different periods of starvation and used their ileum tissue for RNA sequencing analysis. In this study, we aimed to identify lncRNAs that have important functions in starvation stress, analyze their functions, and discover potential molecular targets for alleviating starvation stress, to provide a theoretical reference for subsequent in-depth research.

## MATERIALS AND METHODS

### Ethics statement

This research strictly adhered to the principles of animal use prescribed by the China Laboratory Animal Science Association. The study was approved by the Animal Ethics Committee of Shanxi Agricultural University (Taigu, China). The approval number for the Ethics Committee agreement was SXAU-EAW-2018P002005. The animals were humanely sacrificed as necessary to ameliorate suffering.

### Sample collection

Yorkshire piglets were bred at the animal experiment station of Shanxi Agricultural University in accordance with the National Research Council (NRC) [16]. The test animals were from three litters of half-sibling piglets of the same birth age. On the weaning day, three boars with similar body weights (25 d-old, 5.96±0.15 kg) were selected from each litter, and nine weaned Yorkshire piglets were divided into three groups according to the block design principle. The piglets were randomly divided into a long-term starvation stress group (72 h, abbreviated as IHT-72), short-term starvation stress group (48 h, abbreviated as IHT-48), and a normal group (control group, abbreviated as ICT). The piglets were sacrificed, and ileum samples, which connected to the ileocecal ligament, were collected. The samples were washed with phosphate-buffered saline and immediately frozen in liquid nitrogen until RNA extraction.

### Morphological observation of ileum villi

The ileum samples (ICT, IHT-48, and IHT-72) were analyzed following hematoxylin and eosin (H&E) staining (Bosterbio, Wuhan, Hubei, China). The tissue samples were fixed in 4% paraformaldehyde for 24 h and processed using routine histological methods. Subsequently, 7 μm-thick sections were cut using a Leica RM2265 (Leica, Wetzlar, Germany) and stained. Three images were acquired (Olympus, Tokyo, Japan) for each section, and three sections were selected for each piglet. The lengths of intestinal villi were calculated and counted using SPSS ver.22.0 (IBM Corp., Armonk, NY, USA).

### RNA extraction, library preparation, and high-throughput sequencing

Total RNA was extracted from the nine libraries using TRIzol reagent (Invitrogen, Carlsbad, CA, USA) according to the manufacturer’s instructions. RNA integrity was detected by performing 1% agarose gel electrophoresis, and the purity and concentration were determined using an ND-2000 nucleic acid-protein analyzer (Nanodrop Technologies, Winooski, VT, USA). The ratio of absorbances at 260 nm and 280 nm was determined, with values ranging from 1.8 to 2.0, indicating an RNA concentration of approximately 1,000 ng/μL. After total RNA was extracted, the rRNAs were removed to retain mRNAs and ncRNAs. The cDNA library was generated according to the instructions of the TruSeqRapid Duo cBot Sample Loading kit (Illumina, San Diego, CA, USA) and sequenced using an Illumina HiSeqTM 4000 by Gene Denovo Biotechnology Co. (Guangzhou, China).

### Transcriptome assembly

To obtain high-quality clean reads, reads were filtered using fastp [17] (version 0.18.0). Data were cleaned by removing reads containing adapters, those containing more than 10% poly(N), and low-quality reads (containing more than 50% low-quality [Q-value≤20] bases) from the raw data. The short-read sequence alignment tool Bowtie2 [18] (version 2.2.8) was used to map the read sequence to the ribosomal RNA (rRNA) database. An index of the reference genome (Sscrofa11.1 [GCF_000003025.6]) was built, and paired-end clean reads were mapped to the reference genome using HISAT2 (version 2.1.0). The rRNA database uses the Nucleotide Sequence Database. Transcripts were reconstructed using Stringtie software (version 1.3.4), which together with HISAT2 allowed us to identify new genes and new splice variants of known genes.

### Identification and annotation of novel lncRNAs

The lncRNAs were screened according to a previously described process (SI Appendix, Figure S1). From the remaining transcripts that overlapped (>1 bp) with pig protein-coding genes, transcripts <200 bp and single-exon transcripts were removed. The coding potential calculator (CPC) (version 0.9-r2) and Coding-Non-Coding-Index (CNCI) (version 2) tools were used to assess the coding potential of the remaining transcripts, and transcripts with a CPC score >0 and CNCI score >0 were removed. The intersection of both non-protein-coding potential results was considered lncRNAs.

### Differential expression analysis of lncRNAs

Differential expression of RNA and lncRNAs among different groups was analyzed using DESeq2 software. In addition, different samples in the same group were compared with edgeR. Transcripts with a false discovery rate (FDR) less than 0.05 and an absolute fold change ≥2 were considered differentially expressed. Differentially expressed coding RNAs were subjected to enrichment analysis based on gene ontology (GO) functions and Kyoto encyclopedia of genes and genomes (KEGG) pathway analysis.

### Bioinformatics analysis

Sequence alignment, location, and sequence feature analyses were conducted using the NCBI (https://www.ncbi.nlm.nih.gov), Ensembl (http://asia.ensembl.org/index.html), and UCSC (https://genome.ucsc.edu) websites. GO and KEGG pathway enrichment analyses were conducted using DAVID (http://david.niaid.nih.gov). We performed gene set enrichment analysis (GSEA) using GSEA [19] and MSigDB [19] software to identify whether a set of genes in specific pathways was associated with significant differences between two groups.

### LncRNA–mRNA association analysis

lncRNAs regulate mRNA expression levels via cis and trans interactions. Target genes within 10 kb of differentially expressed lncRNAs (DElncRNA) were selected to explore cis-regulation, and DElncRNA–mRNA target gene pairs with a Pearson correlation coefficient greater than 0.99 were selected to explore co-expression regulation. DElncRNA–mRNAs were visualized using Cytoscape v3.8.2.

### Gene expression detection by real time quantitative polymerase chain reaction

The extracted total RNA was diluted to 500 ng/μL and reverse-transcribed according to the instructions of the PrimeScript RT reagent kit with gDNA Eraser (TaKaRa Bio, Dalian, China). real time quantitative polymerase chain reaction (RT-qPCR) was performed on a CFX96 Real-Time PCR detection system (Bio-Rad, Hercules, CA, USA) using a SYBR Green RT-qPCR kit (TaKaRa Bio, China). 18S rRNA was used as an internal reference gene, and the sequences of primers used are listed in Supplementary Table S1. Three technical replicates were performed for each sample, along with a non-template negative control and a negative control without reverse transcriptase. The RT-qPCR cycling parameters were as follows: pre-denaturation at 95°C for 30 s, followed by 40 cycles of denaturation at 95°C for 30 s and annealing/extension at 60°C for 30 s. Melting curve analysis comprised a denaturation step at 95°C for 30 s, 60°C for 1 min, and 95°C for 30 s.

### Statistical analyses

RT-qPCR results were used to calculate the relative expression levels of the target genes using the 2^−ΔΔCt^ method. Statistical analyses were performed using SPSS ver.22.0 (IBM Corp., USA). Differences in gene expression among the different groups were identified using analysis of variance using Duncan’s test for significance. Pearson correlation analysis was used to determine the relationship between lncRNA expression and mRNA expression and to verify RNA-seq and RT-qPCR results. Graphpad version 8.0 (GraphPad Software, San Diego, CA, USA) was used to draw histograms. All tests were two-tailed, and a p-value <0.05 was considered a significant difference.

### Data availability

The sequencing data obtained have been deposited in the Sequence Read Archive with the accession number PRJNA 770271.

## RESULTS

### Morphological examination of ileum in piglets with starvation stress

**Figure 1:**
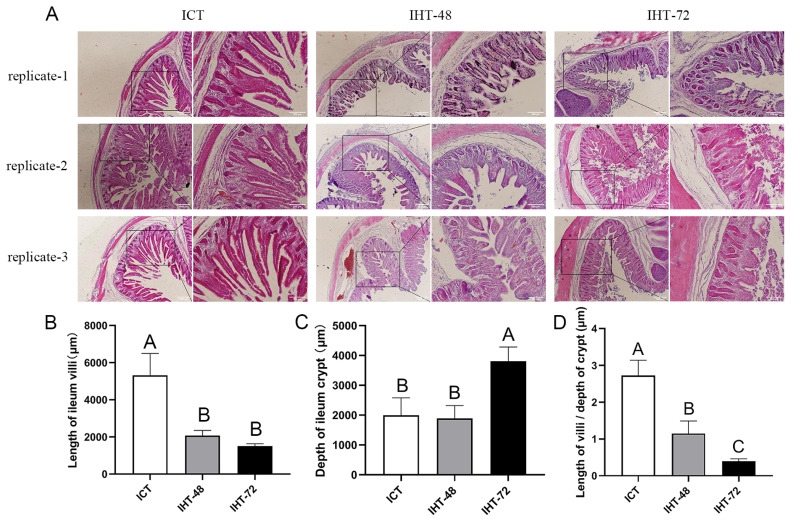
(A) Histological analysis of *Sus scrofa* ileum under starvation stress. ICT is the control, IHT-48 indicates piglets subjected to starvation stress for 48 h, and IHT-72 is piglets subjected to starvation stress for 72 h. (B-D) Histogram comparing the length of the villi, depth of the crypt, and villi/crypt ratio in the ileum among the two treatments and the control (^A–C^ indicates p<0.01).

The morphological comparison of the piglet ilea under different treatments (Figure 1A) revealed that an increase in starvation time corresponded with shortening of the ileal villi (Figure 1B) and increases in the crypt depth (Figure 1C) and ratio of villus height to crypt depth (Figure 1D). In summary, as the starvation stress time increased, the ileum tissue damage increased.

### Identification of lncRNAs in ileum of piglets

**Figure 2:**
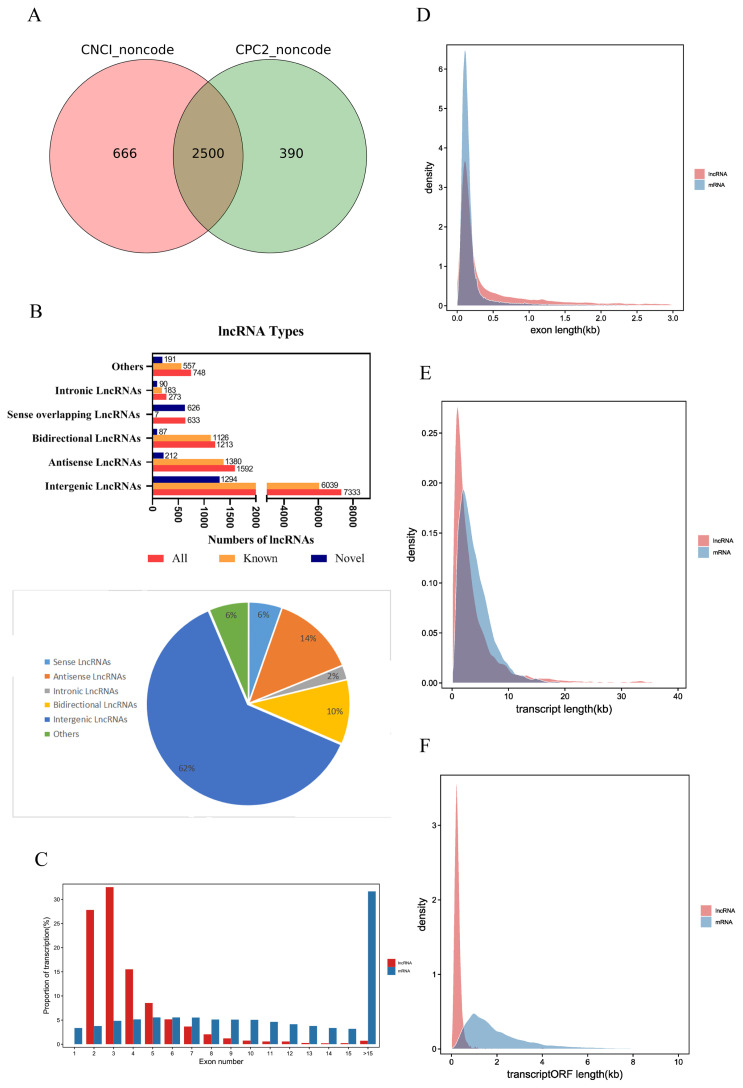
Identification and expression profile analysis of lncRNAs obtained from *Sus scrofa* ileum under starvation stress for 48 h and 72 h and control conditions. (A) Novel lncRNA-coding ability prediction. (B) Type of lncRNAs. (C) Number of lncRNA and mRNA exons. (D) Lengths of lncRNA and mRNA exons. (E) Length of lncRNA and mRNA transcripts. (F) Length of lncRNA and mRNA transcript ORFs. ORF, open reading frames.

The raw reads of each sample were between 11.1 and 14.7 billion. After excluding the aptamer sequence and low-quality reads, 773,785,766 clean reads were obtained from the nine RNA-Seq datasets (Table 1). The Q30 of each sample was ≥91.96%, the N percentage was 0.00%, and the guanine-cytosine (GC) percentage of each library was between 44.20% and 46.22%. These data demonstrated that the sequencing had high reliability. After removing the rRNA reads from the alignment, they were compared to the pig reference genome, and 96.67% to 95.03% reads were successfully mapped. Stringties was used to reconstruct transcripts, which were compared with the known transcripts recorded in the Ensembl database. Subsequently, 11,792 lncRNAs and 63,682 mRNAs were obtained, including 2,500 novel lncRNAs and 69 novel mRNAs (SI Appendix, Table S1). We determined the coding potential of the novel lncRNAs using the prediction software CNCI and CPC2 (Figure 2A).

### LncRNAs expression profile analysis

According to the relative position of lncRNAs and protein-coding genes in the genome, lncRNAs can be divided into five categories, intergenic lncRNAs, bidirectional lncRNAs, intronic lncRNAs, antisense lncRNAs, and sense overlapping lncRNAs. Among them, intergenic lncRNAs were the most common in our study, with 7,333 accounting for approximately 62% of lncRNAs. Intronic lncRNAs were the least abundant, with a total of 272, accounting for approximately 2% of lncRNAs (Figure 2B).

The average number of exons in lncRNAs was 3.90±2.65, which was significantly less than the average number of exons in mRNAs (13.66±11.64) (Figure 2C). The average exon length in lncRNAs was 1.03±2.46 kb, which was significantly longer than the exon length in mRNAs (0.31±0.80 kb) (Figure 2D). The average transcript length of lncRNAs was 4.01± 5.62 kb, which was shorter than the transcript length of mRNAs (4.23±3.15 kb) (Figure 2E). The average open reading frame (ORF) length of lncRNAs was 0.29±0.30 kb, which was significantly shorter than the ORF length of mRNAs (2.15±1.99 kb) (Figure 2F). These results indicate that piglet lncRNAs have fewer but longer exons, longer transcripts, and longer ORFs than mRNA transcripts.

### Sample correlation analysis

**Figure 3:**
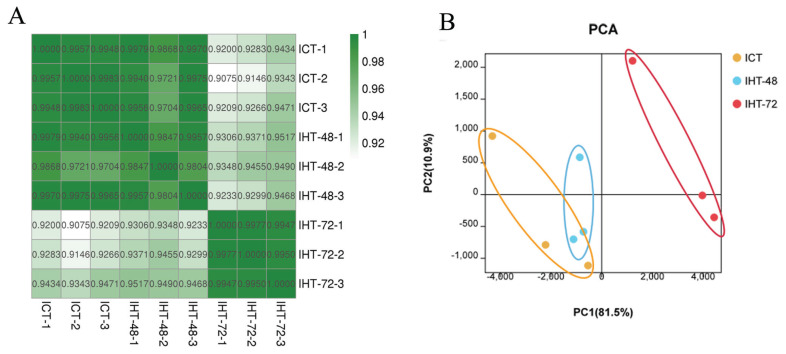
Sample correlation analysis of *Sus scrofa* ileum under starvation stress. ICT is the control, IHT-48 indicates piglets subjected to starvation stress for 48 h, and IHT-72 is piglets subjected to starvation stress for 72 h. (A) Correlation heat map. (B) PCA analysis. PCA, principal component analysis.

To assess the reliability and operational stability of the test results, we performed a Pearson analysis of lncRNA expression in each sample. The correlation coefficients between the samples were between 0.9804 and 0.9977 (SI Appendix, Figure S2). The samples in each treatment group showed a very high correlation with each other. Among them, the correlation between the replicates within the ICT and IHT-72 groups was greater than 0.99. However, the second sample in the IHT-48 group had a low correlation with the other two samples, although both values were greater than 0.98. A comparison of the correlation between groups found that ICT and IHT-48 groups had a better correlation, and both of these treatment groups had a weaker correlation with the IHT-72 group (Figure 3A). In the principal component analysis (PCA), the ICT and IHT-48 groups were located relatively closely but were situated relatively far from the IHT-72 group (Figure 3B). This indicates that differences in the ileum can be observed after 72 h of starvation stress.

### Characteristics of DElncRNAs and differentially expressed mRNAs (DEmRNAs)

**Figure 4:**
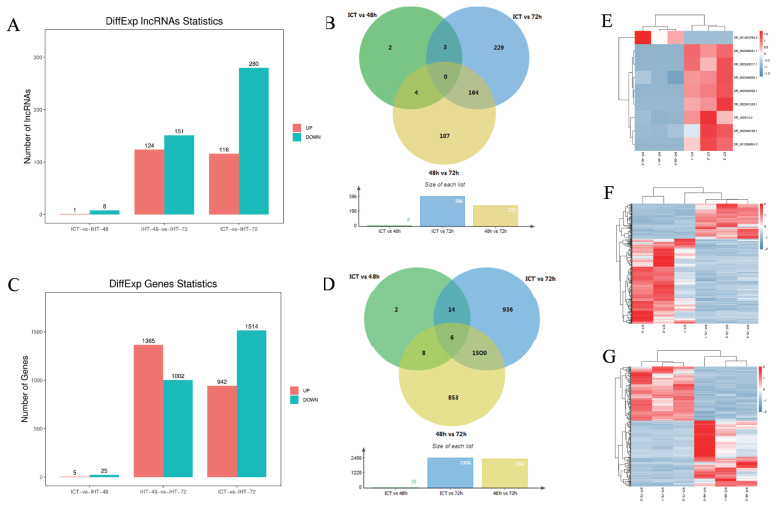
Analysis of differentially expressed (DE)lncRNAs and DEmRNAs of *Sus scrofa* ileum under starvation stress. ICT is the control, IHT-48 is piglets subjected to starvation stress for 48 h, and IHT-72 is piglets subjected to starvation stress for 72 h. (A) The bar graph shows the number of DElncRNAs. (B) Venn diagram showing DElncRNAs. (C) The bar graph shows the number of DEmRNAs. (D) Venn diagram of DEmRNAs. (E) Heat map of differentially expressed genes in ICT group and IHT-48 group (z-scores). (F) Heat map of differentially expressed genes in IHT-48 group and IHT-72 group (z-scores). (G) Heat map of differentially expressed genes in ICT group and IHT-72 group (z-scores).

The fragments per kilobase of transcript per million mapped reads (FKPM) values of lncRNAs (average 2.76) were significantly lower than those of the coding genes (average 14.82), including 23 highly expressed lncRNAs (over 100) and 259 moderately expressed lncRNAs (between 10 and 100). Based on an FDR of 5% and a q-value <0.05, 509 DElncRNAs (Figure 4A, 4B) and 3,319 DEmRNAs (Figure 4C, 4D) were identified by pairwise comparisons among the three treatment groups. There were nine DElncRNAs between ICT and IHT-48, among which, the expression level of one was upregulated, whereas those of eight were downregulated (Figure 4E). There were 275 DElncRNAs between IHT-48 and IHT-72, of which, the expression levels of 24 were upregulated and those of 151 were downregulated (Figure 4F). There were 396 DElncRNAs between ICT and IHT-72, of which, expression levels of 116 were upregulated and those of 280 were downregulated (Figure 4G). The trends in lncRNAs and mRNAs were similar. A Venn diagram showed that DElncRNAs and DEmRNAs were mainly concentrated in IHT-48 vs IHT-72 and ICT vs IHT-72 comparisons (Figure 4B, 4D), indicating that the piglet ileum underwent significant changes after 72 h of starvation. These results were verified by GSEA of DEmRNAs. Compared to the control animals and animals subjected to 48 h of starvation, animals subjected to 72 h of starvation showed decreased metabolism. This decrease affected pathways such as glycolysis, gluconeogenesis, the tricarboxylic acid cycle (TCA cycle), and fructose and mannose metabolic pathways. It also impacted pyruvate metabolism, as well as vitamin digestion and absorption. Furthermore, this treatment increased inflammation-related cells and diseases, such as cell adhesion molecules (CAMs), Th1/Th2 cell differentiation, inflammatory bowel disease, and malaria (SI Appendix, Figure S3).

### Potential cis-regulatory mechanisms between porcine DElncRNAs and neighboring mRNAs

**Figure 5:**
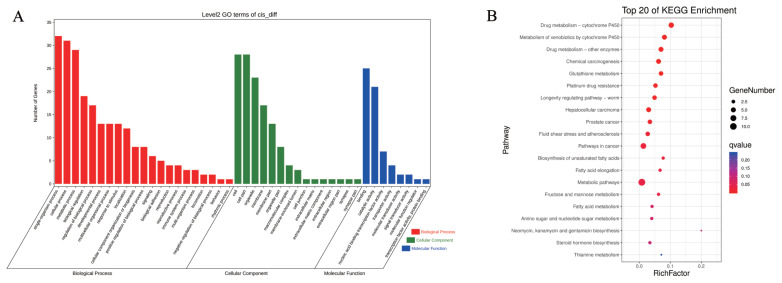
Potential cis-regulatory interaction between differentially expressed (DE)lncRNA and neighboring mRNAs in *Sus scrofa* ileum under starvation stress. (A) GO analysis. (B) KEGG analysis. GO, gene ontology; KEGG, Kyoto encyclopedia of genes and genomes.

Numerous studies have indicated that lncRNAs exert either positive or negative effects to regulate the expression of neighboring protein-coding genes [20,21]. Therefore, we extracted the information related to the location of differential lncRNAs from the transcription file and porcine genome database (http://genome-asia.ucsc.edu ) and selected mRNAs located within 10 kb upstream or downstream of these lncRNAs. We screened 59 DElncRNAs and 57 DEmRNAs and obtained 64 pairs of DElncRNA–DEmRNAs (SI Appendix, Table S2). GO analysis revealed that the DElncRNAs–DEmRNAs were enriched in BPs, cell components (CCs), and molecular functions (MFs). Among these, 21 BPs, 15 CCs, and eight MFs were involved (Figure 5A), and the proportion of genes enriched in terms such as metabolic processes, developmental processes, cell parts, catalytic activity, and nucleic acid binding was higher. To clarify the specific signaling pathways affected by the targets, KEGG analysis was conducted. KEGG analysis results showed that the target genes were enriched in 97 pathways (Figure 5B). Among the top 20 significantly enriched pathways, those related to energy metabolism were most enriched. These included seven items including metabolic pathways, fructose and mannose metabolism, and fatty acid metabolism, among others. The pathways related to drug absorption and metabolism, including drug metabolism - cytochrome P450, drug metabolism by other enzymes, and chemical carcinogenesis were the second most abundant. Four disease-related pathways, namely involved in hepatocellular carcinoma, prostate cancer, fluid shear stress in atherosclerosis, and cancer were also enriched. The cis-regulation analysis showed that after the piglets were subjected to starvation stress, significant changes occurred in energy homeostasis, metabolism, drug absorption, and disease occurrence.

### Potential trans-regulatory mechanisms between porcine DElncRNAs and their co-expressed mRNAs

**Figure 6:**
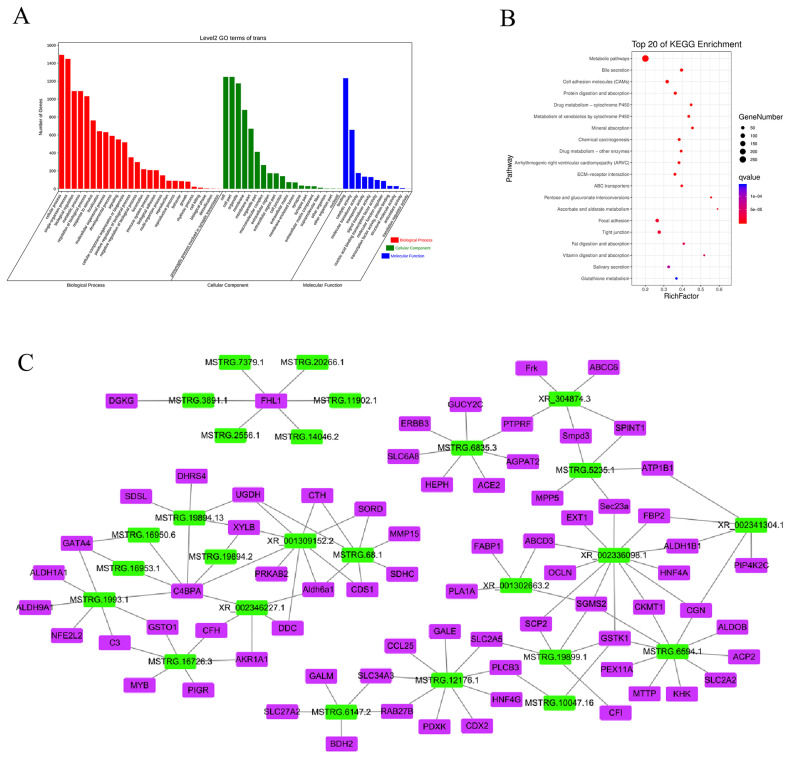
Potential trans-regulatory interactions between differentially expressed (DE)lncRNA and co-expressed mRNAs in *Sus scrofa* ileum under starvation stress. (A) GO analysis. (B) KEGG analysis. (C) Co-expression network analysis. GO, gene ontology; KEGG, Kyoto encyclopedia of genes and genomes.

In trans-regulation, the function of lncRNA is not linked to the mRNA location but to the co-expressed mRNA. We screened 447 DElncRNAs and 2,601 DEmRNAs and obtained 38,265 pairs of DElncRNAs–DEmRNAs. GO analysis revealed that 26 BPs were involved, and 19 CC sand 11 MFs were enriched (Figure 6A). Similar to the cis-regulated GO results, these DElncRNAs were determined to have important functions in regulating metabolic processes, cellular components, nucleic acid binding, and transcription factor activity. KEGG analysis showed that the target genes were enriched in 328 pathways (Figure 6B). Most of the top 20 pathways with significant enrichment were related to energy metabolism, including metabolic pathways, protein digestion and absorption, mineral absorption, fat digestion and absorption, and vitamin digestion and absorption, among others. However, enrichment was also observed in other pathways, such as drug metabolism-cytochrome P450, arrhythmogenic right ventricular cardiomyopathy, and tight junctions. These enrichment results were similar to the enrichment results of cis-regulation, indicating that the piglets exhibited significant changes to their metabolism and susceptibility to disease during starvation stress.

Cytoscape was used to map DElncRNAs/DEmRNAs with a Pearson correlation coefficient absolute value greater than 0.99 (p<0.01; SI Appendix, Figure S4). The results showed a total of 1,000 interactions among 212 DElncRNAs and 599 DEmRNAs. There were 998 positive interactions (red lines) and two negative (black lines) interactions among pairs within the network, and most DElncRNA–DEmRNA pairs were positively correlated. To more clearly identify lncRNAs that play a key role in hunger stress, 121 pairs of DElncRNAs–DEmRNAs with both GO and KEGG enrichment results and ranking in the top 15 in node number were selected (including 26 DElncRNAs and 72 DEmRNAs), as shown in Figure 6C. All of these networks had positive interactions (red lines). Among them, lncRNA XR_002336098.1 had the most co-expressed mRNAs (12 mRNAs), whereas complement component 4 binding protein alpha (*C4BPA*) mRNA had the most co-expressed lncRNAs (7 lncRNAs). The functions of DElncRNAs and DEmRNAs in the interaction network were mainly enriched in metabolism and disease. For example, MSTRG.19894.13 participates in metabolism by regulating the expression of serine dehydratase like (*SDSL*), UDP-glucose 6-dehydrogenase (*UGDH*), and xylulokinase (*XYLB*), among other genes, and MSTRG.16726.3 participates in human diseases by regulating the expression of complement C3 (*C3*), complement factor H (*CFH*), and glutathione S-transferase omega 1 (*GSTO1*), among other genes. In the interaction network, MSTRG.12176.1 had more interactive pairs and was expressed at higher levels, which suggested that it might be the key lncRNA in the ileum of piglets subjected to long-term starvation stress. Further, it regulates metabolism by modulating the expression of genes such as UDP-galactose-4-epimerase (*GALE*) and pyridoxal kinase (*PDXK*) and can participate in human diseases by regulating the expression of genes such as caudal type homeobox 2 (*CDX2*) and phospholipase C beta 3 (*PLCB3*).

### Accuracy of RT-qPCR verification results

**Figure 7:**
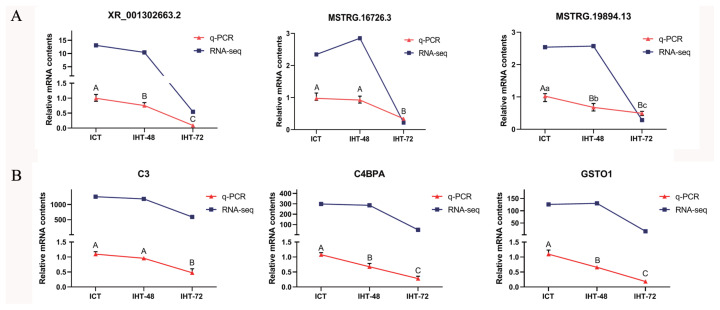
Accuracy of RT-qPCR verification results for the lncRNAs and mRNAs of *Sus scrofa* ileum under starvation stress. (A) RT-qPCR verification of lncRNAs. (B) RT-qPCR verification of mRNAs. ^a–c^ Indicates p<0.05, ^A–C^ indicates p<0.01.

To verify the reliability of the RNA sequencing data, three DElncRNAs (XR_001302663.2, MSTRG.16726.3, and MSTRG.19894.13; Figure. 7A) and three DEmRNAs (*C3*, *C4BPA*, and *GSTO1*; Figure 7B) were randomly selected. Based on NCBI (https://www.ncbi.nlm.nih.gov) and UCSC (https://genome.ucsc.edu) reference sequences, primers were designed using Primer 5.0 to validate these lncRNAs and mRNAs (SI Appendix, Table S3). The RT-qPCR results showed that the expression profiles of XR_001302663.2, *C3*, *C4BPA*, and *GSTO1* were consistent with the sequencing results, and the expression profiles of MSTRG.16726.3 and MSTRG.19894.13 were similar to the sequencing results (Figure 7A). The expression levels of these lncRNAs and mRNAs were comparable in ICT and IHT-48 groups and were significantly higher than those in the IHT-72 group.

## DISCUSSION

Despite the continuous increase in food production in modern times, starvation continues in various parts of the world either due to poverty or geological disasters. In contrast, several individuals starve voluntarily; for example, more people are inclined to adopt improper ways to lose weight by fasting for long durations [3]. The pig was used in this experiment as an ideal model to study human metabolic diseases [15]. In this study, we analyzed the transcriptional changes in piglets subjected to starvation stress via high-throughput sequencing and revealed the damage caused by starvation stress to the body from the perspective of lncRNA expression.

Genome-wide studies have revealed that lncRNAs play an important role in pigs [22]. However, few studies have been conducted on the expression of starvation stress-related lncRNA expression in pigs. In this study, we established a piglet model of starvation stress and found that the intestine is one of the key tissues damaged by starvation stress. Illumina HiSeqTM 4000 high-throughput sequencing was used to construct differential expression profiles of starvation stress-related lncRNAs in the piglet ileum. By testing the relationships between the samples, we found that at the transcriptome level, negligible differences were observed between the control group and the group starved for 48 h. However, compared with the ICT and IHT-48 groups, notable differences were observed in the group starved for 72 h. In our study, 72 h starvation inhibited glycolysis, gluconeogenesis, the TCA cycle, fructose and mannose metabolism, pyruvate metabolism, and vitamin digestion and absorption. This result suggests that long-term starvation leads to an insufficient energy supply, exhaustion of glycogen storage, and nutrient deficits. Furthermore, long-term starvation affects the body’s immunity and increases the risk of disease. During 72 h of starvation stress, mRNAs related to CAMs were overexpressed. The CAMs play an important role in the occurrence of diseases and are closely related to inflammation [23]. Further, 72 h starvation could induce Th1/Th2 cell differentiation and lead to inflammatory bowel disease and malaria. However, it might reduce the onset of diabetes, which could be related to the insufficient energy supply. In summary, prolonged starvation can cause irreversible damage to the body and at the same time provides a theoretical basis for the “golden 72 hours” associated with earthquake rescue.

A co-expression sub-network containing 26 DElncRNAs and 72 DEmRNAs was reconstructed to reveal the mechanisms underlying starvation stress and its effect. LncRNA MSTRG.19894.13 was determined to be an important lncRNA in the constructed network. *C4BPA*, dehydrogenase/reductase 4 (*DHRS4*), *SDSL*, *UGDH*, and *XYLB* were identified as the target genes of this lncRNA. *C4BPA* controls the classic complement activation pathway and interacts with RelA, a member of the nuclear factor kappa-B (NF-κB) family [24]. The expression of *C4BPA* is regulated by stress [24]. *DHRS4* is a peroxisomal member of the short-chain dehydrogenase/reductase superfamily. Unlike that in its human counterpart, a mutation at Thr177 with the corresponding residue Asn stabilizes DHRS4 in pigs and prevents its cold-induced inactivation [25]. SDSL, also known as SDH2, exhibits low serine dehydratase and threonine dehydratase activities [26]. UGDH is a cytosolic enzyme that catalyzes the oxidation of UDP-glucose to UDP-glucuronic acid, thereby participating in the biosynthesis of glycosaminoglycans, such as hyaluronic acid, chondroitin sulfate, and heparan sulfate [27]. In patients with lung adenocarcinoma, the presence of UGDH in the nucleus is correlated with poorly differentiated cells and larger tumors and could indicate overall reduced survival [28]. *XYLB* encodes a xylulose kinase-like protein, and its forced overexpression is an effective strategy to improve xylose utilization and P(3HB) production in *Burkholderia sacchari* [29]. Our results showed that MSTRG. 19894.13 regulates the expression of *C4BPA*, *DHRS4*, *SDSL*, *UGDH*, and *XYLB*, and therefore, it might play an important role in regulating functions such as complement activation, enzyme activity, biosynthesis, and energy utilization.

The co-expressed target genes of MSTRG.16726.3 were determined to be aldo-keto reductase family 1 member A1 (*AKR1A1*), *C3*, *CFH*, *GST01*, MYB proto-oncogene (*MYB*), and polymeric immunoglobulin receptor (*PIGR*). Studies have shown that AKR1A1 can synthesize ascorbic acid in rodents [30] and is involved in the metabolism of gamma-hydroxybutyric acid in human liver cancer-derived HepG2 cells [30]. In contrast, C3 plays a central role in activation of the complement system and can be used as a biomarker for insulin resistance and cardiometabolic diseases [31]. CFH plays an important role in regulating complement activation and limits the effect of innate defense mechanisms against microbial infection [32]. GSTO1 is involved in the metabolism of xenobiotics and carcinogens, such as with cutaneous malignant melanoma [33]. Mutations in and the overexpression of *MYB* were first discovered in leukemia cells and were recently discovered in solid cancers [34]. *MYB* plays an important role in hematopoietic functions and tumorigenesis [34]. The circ-XPO4 of the small extracellular vesicles present in milk promotes the expression of *PIGR* in intestinal cells by inhibiting miR-221-5p, thereby increasing the level of intestinal SIgA [35]. In conclusion, MSTRG.16726.3 might play an important role in complement activation, the metabolism of exogenous and carcinogenic substances, resistance against pathogenic microorganisms, and improvements in intestinal immunity.

The following nine genes are targeted by MSTRG.12176.1: C-C motif chemokine ligand 25 (*CCL25*), *CDX2*, *GALE*, hepatocyte nuclear factor 4 gamma (*HNF4G*), *PDXK*, *PLCB3*, *RAB27B*, solute carrier family 2 member 5 (*SLC2A5*), and solute carrier family 34 member 3 (*SLC34A3*). CCL25, a member of the chemokine CC subfamily, has also been recognized as a thymus-expressed chemokine [36]. CCL25 and its receptor C-C motif chemokine receptor 9 (CCR9) are overexpressed in cancers such as leukemia, ovarian cancer, breast cancer, prostate cancer, liver cancer, lung cancer, and melanoma [36]. *CDX2* is a major regulator of intestine-specific genes involved in cell growth and differentiation [37]. Decreased expression of *CDX2* is associated with mucinous tumors, lymph node involvement, and high-grade tumors [37]. *GALE* encodes UDP-galactose-4-epimerase, and mutations in *GALE* result in epimerase-deficient galactosemia, also referred to as galactosemia type 3, a disease characterized by early onset cataracts, liver damage, deafness, and mental retardation [38]. *HNF4G* is overexpressed in colorectal cancer (CRC) and promotes CRC cell proliferation via the PI3K/AKT pathway by targeting G protein subunit gamma 12 (GNG12) and protein tyrosine kinase 2 (PTK2) [39]. *PDXK* encodes a pyridoxal kinase, which converts inactive B vitamins to the active cofactor pyridoxal 5′-phosphate (PLP) [40]. Hereditary polyneuropathy with optic atrophy can occur due to a *PDXK* variant leading to impaired vitamin B6 metabolism [40]. *PLCB3* is involved in innate immunity, and studies have shown that silencing *PLCB3* enhances the inflammatory signaling cascade of toll-like receptors [41]. *RAB27B* is an RAS oncogenic family member gene that regulates extracellular vesicle production in cells infected with Kaposi’s sarcoma-associated herpesvirus to promote cell survival and persistent infection [42]. The fructose transporter encoded by *SLC2A5* is required for intestinal fructose absorption [43]. *SLC2A5* expression is induced in the intestine and skeletal muscle of patients with type 2 diabetes and in certain cancers dependent on fructose metabolism [43]. In renal brush border cells, *SLC34A3* is involved in transporting intracellular phosphate via sodium co-transport and contributes to the maintenance of inorganic phosphate concentrations in the kidneys [44]. Mutations in *SLC34A3* can cause hereditary hypophosphatemic rickets with hypercalciuria [44]. Therefore, by acting on multiple target genes, MSTRG.12176.1 can play an important role in the starvation stress in piglets and affect energy metabolism, nutrient absorption, and disease occurrence and development.

In this study, 11,792 lncRNAs and 63,682 mRNAs were identified through transcriptome sequencing of ileum tissue obtained from pigs subjected to starvation stress. These included 2,500 novel lncRNAs, 509 DElncRNAs, and 3,319 DEmRNAs. The target genes of DElncRNAs were determined to be mainly involved in metabolic pathways, cellular processes, immune system processes, digestive systems, and transport activities, among others. A co-expression network induced by starvation stress in pigs was constructed, and the key lncRNAs were analyzed. However, the specific mechanism by which these lncRNAs regulate metabolism is unknown. Further studies are required to elucidate the molecular mechanisms linking lncRNAs to the regulation of starvation stress in pigs.

## Figures and Tables

**Table 1:** Base information before and after filtering and statistics of comparison analysis

Sample	Before filter				After filter				
	
	Raw data (bp)	Q30 (%)	N (%)	GC (%)	Clean data (bp)	Q30 (%)	N (%)	GC (%)	Total mapped (%)
ICT-1	14,619 702,300	13,644,665,568 (93.33)	18,498 (0.00)	6,670,799,401 (45.63)	14,527 873,806	13,572,090,462 (93.42)	17,340 (0.00)	6,620,560,805 (45.57)	93,852,426 (96.54)
ICT-2	12,638 687,400	11,802,361,425 (93.38)	15,883 (0.00)	5,641,122,748 (44.63)	12,560 290,461	11,739,709,800 (93.47)	14,835 (0.00)	5,598,601,151 (44.57)	81,275,360 (96.67)
ICT-3	13,976 345,700	13,052,331,529 (93.39)	20,203 (0.00)	6,299,955,597 (45.08)	13,897 008,834	12,989,976,631 (93.47)	19,860 (0.00)	6,256,759,706 (45.02)	89,519,813 (96.29)
IHT-48-1	13,107 414,300	12,149,829,392 (92.69)	18,775 (0.00)	5,801,312,645 (44.26)	13,027 940,166	12,087,840,185 (92.78)	18,494 (0.00)	5,757,706,331 (44.20)	83,794,100 (96.15)
IHT-48-2	11,361 532,800	10,560,771,310 (92.95)	137,002 (0.00)	5,256,821,028 (46.27)	11,292 380,797	10,507,231,870 (93.05)	50,960 (0.00)	5,219,261,006 (46.22)	72,399,012 (95.90)
IHT-48-3	12,832 586,700	11,973,715,965 (93.31)	379,755 (0.00)	5,750,696,421 (44.81)	12,748 182,544	11,908,250,713 (93.41)	105,400 (0.00)	5,705,324,400 (44.75)	81,556,124 (95.60)
IHT-72-1	11,778 739,200	10,874,124,989 (92.32)	19,660 (0.00)	5,242,309,705 (44.51)	11,715 423,009	10,829,303,843 (92.44)	19,545 (0.00)	5,207,794,628 (44.45)	74,673,381 (95.40)
IHT-72-2	11,322 221,700	10,478,162,856 (92.55)	18,684 (0.00)	5,041,842,046 (44.53)	11,260 120,502	10,433,540,423 (92.66)	18,589 (0.00)	5,008,169,056 (44.48)	71,746,932 (95.33)
IHT-72-3	14,693 745,900	13,492,497,483 (91.82)	24,109 (0.00)	6,533,447,306 (44.46)	14,582 740,828	13,410,149,381 (91.96)	23,941 (0.00)	6,472,995,836 (44.39)	92,752,279 (95.03)

GC, guanine-cytosine.

## SUPPLEMENTARY MATERIAL

**SI Appendix, Figure S1**. Screening process for lncRNAs. **SI Appendix, Figure S2**. Pearson correlation analysis between samples. **SI Appendix, Figure S3**. GSEA enrichment analysis. **SI Appendix, Table S1**. The transcript information statistics of ileum tissue of starvation-stressed pigs **SI Appendix, Table S2**. Potential cis-regulation between porcine DELncRNA and its neighboring mRNAs **SI Appendix, Table S3**. Primers used in this study
