# Supplementary material for: Long non-coding RNAs in Sus scrofa ileum under starvation stress
Source: Anim Biosci. 2022 Mar 2;35(7):975–88. doi: 10.5713/ab.21.0483 (PMC9271390; doi:10.5713/ab.21.0483)
Supplement: Supplementary file 1 [file ab-21-0483-suppl.pdf]

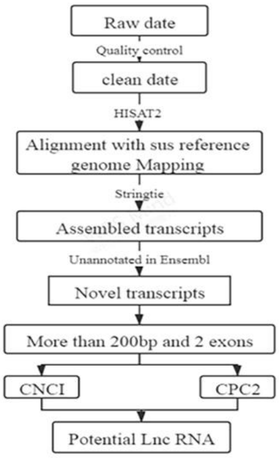

SI Appendix Figure. S1 Screening process for lncRNAs.

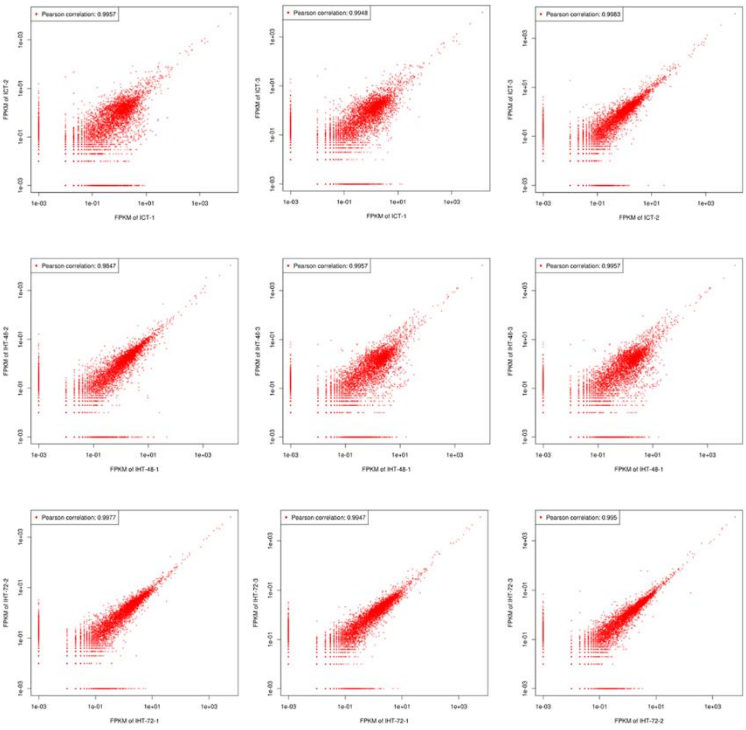

SI Appendix Figure. S2 Pearson correlation analysis between samples.

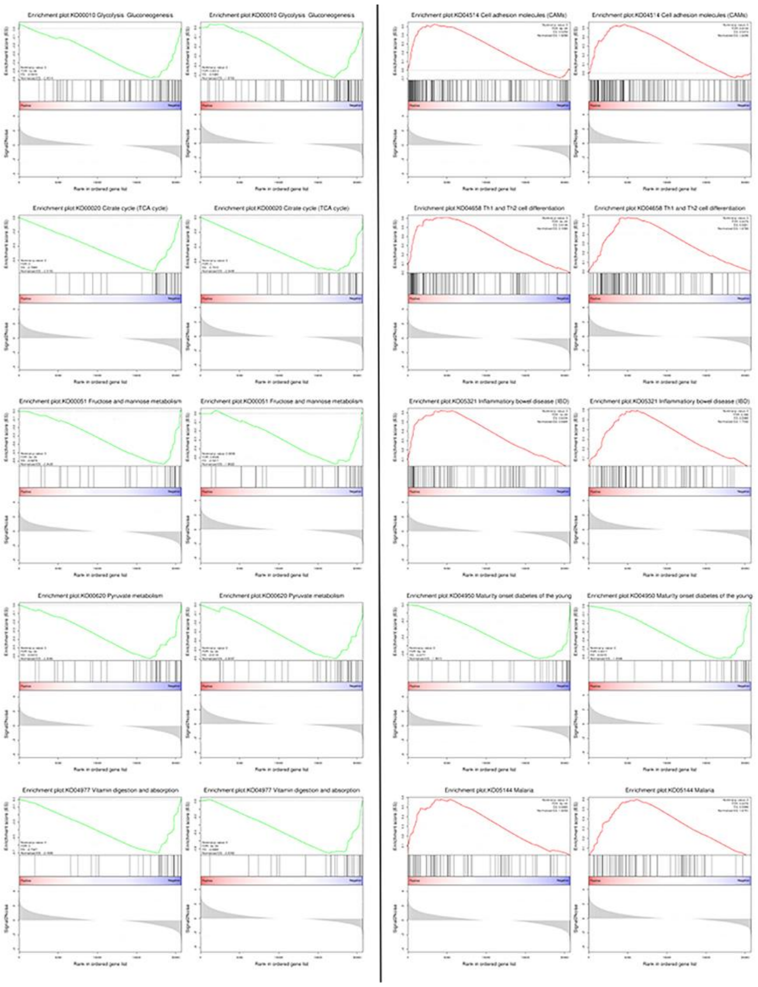

SI Appendix Figure. S3 GSEA enrichment analysis.

SI Appendix, Table. S1 The transcript information statistics of ileum tissue of starvation-stressed pigs												
**Sample**	**Refer_**	**Sequenced_Refer_**	**Novel_**	**Sequenced_Novel_I**	**Total_In**	**Sequenced_Total_**	**Refer_C**	**Sequenced_Refer_Ge**	**Nove**	**Sequenced_Novel_**	**Total_Ge**	**Sequenced_Total_Ge**
all	9292	5834 (62.79%)	2500	2500 (100.00%)	11792	8334 (70.68%)	20666	17674 (85.52%)	69	69 (100.00%)	20735	17743 (85.57%)
ICT-1	9292	3151 (33.91%)	2500	2419 (96.76%)	11792	5570 (47.24%)	20666	16202 (78.40%)	69	66 (95.65%)	20735	16268 (78.46%)
ICT-2	9292	3100 (33.36%)	2500	2354 (94.16%)	11792	5454 (46.25%)	20666	16064 (77.73%)	69	63 (91.30%)	20735	16127 (77.78%)
ICT-3	9292	3304 (35.56%)	2500	2406 (96.24%)	11792	5710 (48.42%)	20666	16206 (78.42%)	69	66 (95.65%)	20735	16272 (78.48%)
IHT-48-1	9292	3155 (33.95%)	2500	2408 (96.32%)	11792	5563 (47.18%)	20666	16068 (77.75%)	69	66 (95.65%)	20735	16134 (77.81%)
IHT-48-2	9292	2984 (32.11%)	2500	2394 (95.76%)	11792	5378 (45.61%)	20666	15969 (77.27%)	69	65 (94.20%)	20735	16034 (77.33%)
IHT-48-3	9292	3105 (33.42%)	2500	2375 (95.00%)	11792	5480 (46.47%)	20666	15982 (77.33%)	69	67 (97.10%)	20735	16049 (77.40%)
IHT-72-1	9292	3216 (34.61%)	2500	2380 (95.20%)	11792	5596 (47.46%)	20666	16141 (78.10%)	69	62 (89.86%)	20735	16203 (78.14%)
IHT-72-2	9292	3321 (35.74%)	2500	2381 (95.24%)	11792	5702 (48.35%)	20666	16185 (78.32%)	69	63 (91.30%)	20735	16248 (78.36%)
IHT-72-3	9292	3481 (37.46%)	2500	2396 (95.84%)	11792	5877 (49.84%)	20666	16314 (78.94%)	69	66 (95.65%)	20735	16380 (79.00%)

SI Appendix, Table. S2 Potential cis-regulation between porcine DELncRNA and its neighboring mRNAs

lncRNA_ID	lncRNA_start	lncRNA_end	GeneID	Symbol	distance	KEGG_A_cl	KEGG_B_cl	Pathway	K_ID	GO Compon	GO Function	GO Process
XR_00130620	126511244	126513958	ncbi_1005222	SLC28A1	36	-	-	-	-	GO:0016021//	GO:0005337//	GO:0015858//
MSTRG.790.2	126511344	126515243	ncbi_1005222	SLC28A1	136	-	-	-	-	GO:0016021//	GO:0005337//	GO:0015858//
MSTRG.1383	217442059	217492619	ncbi_397003	SLC1A1	415	Organismal S	Nervous sys	ko04724//Glu	K05612;K056	GO:0005886//	GO:0000099//	GO:0000101//
MSTRG.1608	249998800	250003356	ncbi_1006260	CTNNAL1	2087	-	-	-	-	GO:0016020//	GO:0003779//	GO:0007265//
XR_00234222	5002406	5043826	ncbi_1102598	Cyth3	204	Cellular Proc	Transport an	ko04144//End	K18441;K184	GO:0019897//	GO:0005085//	GO:0007155//
XR_00234274	97418214	97572537	ncbi_1005142	HAAO	8218	Metabolism;l	Global and ov	ko01100//Me	K00452;K004	GO:0031988//	GO:0005506//	GO:0006568//
XR_00234367	940767	949860	ncbi_1006200	TSTA3	1086	Metabolism;l	Global and ov	ko01100//Me	K02377;K023	GO:0031988//	GO:0016616//	GO:0016337//
MSTRG.6147	100378145	100394346	ncbi_1001529	FMO5	5400	Metabolism	Xenobiotics	ko00982//Dru	K00485	GO:0031090//	GO:0000166//	GO:0044710//
MSTRG.6149	100442062	100443700	ncbi_1005190	NBPF6	3075	-	-	-	-	-	-	-
XR_00234351	110425166	110434989	ncbi_1102603	GSTM1	5710	Human Disea	Cancers;Can	ko05200//Pat	K00799;K007	GO:0044424//	GO:0003824//	-
XR_00234352	110436589	110439016	ncbi_1102603	GSTM1	2792	Human Disea	Cancers;Can	ko05200//Pat	K00799;K007	GO:0044424//	GO:0003824//	GO:0006790//
XR_00234352	110436589	110439016	ncbi_1102603	GSTM1	1683	Human Disea	Cancers;Can	ko05200//Pat	K00799;K007	GO:0044424//	GO:0003824//	-
XR_00234351	110446531	110454628	ncbi_1102603	GSTM1	365	Human Disea	Cancers;Can	ko05200//Pat	K00799;K007	GO:0044424//	GO:0003824//	-
XR_00234388	8360857	8378448	ncbi_1001554	Fam83f	6118	-	-	-	-	-	-	-
MSTRG.6676	10803901	10811647	ncbi_1001532	CSF2RB	4718	Human Disea	Cancers;Sign	ko05200//Pat	K04738;K047	-	-	GO:0007166//
XR_00234402	34223060	34426708	ncbi_1021654	MYRFL	4543	-	-	-	-	GO:0031224//	GO:0001071//	GO:0010468//
XR_00234403	34223060	34426708	ncbi_1021654	MYRFL	4543	-	-	-	-	GO:0031224//	GO:0001071//	GO:0010468//
MSTRG.7431	78266599	78276758	ncbi_396628	VDR	93	Human Disea	Infectious dis	ko05152//Tub	K08539;K085	GO:0043231//	GO:0004879//	GO:0003006//
XR_00234462	43086118	43089178	ncbi_397307	CEBPA	233	Human Disea	Cancers;Can	ko05200//Pat	K09055;K090	GO:0090575//	GO:0000977//	GO:0000079//
MSTRG.8089	44667682	44672083	ncbi_1007370	Lgi4	3807	-	-	-	-	-	-	GO:0007626//
XR_00234469	51596712	51606208	ncbi_1102610	TRAPPC6A	7215	-	-	-	-	GO:0043231//	-	GO:0006810//
XR_00234469	51596712	51606208	ncbi_1102610	HSD17B12	846	Metabolism;l	Global and ov	ko01100//Me	K10251;K102	-	GO:0003824//	GO:0044710//
MSTRG.9264	107278952	107282565	ncbi_397600	GATA6	143	-	-	-	-	GO:0031981//	GO:0000977//	GO:0001666//
XR_00234562	24321506	24324376	ncbi_1001555	BTN3A3	258	-	-	-	-	GO:0031224//	-	-
MSTRG.1009	26443788	26448458	ncbi_1001531	TINAG	153	-	-	-	-	GO:0044420//	GO:0001871//	GO:0002376//
XR_00234576	46553813	46567596	ncbi_397682	GSTA2	3031	Human Disea	Cancers;Can	ko05200//Pat	K00799;K007	-	GO:0003824//	-
XR_00234576	46553813	46567596	ncbi_1005261	GSTA1	3509	Human Disea	Cancers;Can	ko05200//Pat	K00799;K007	-	GO:0003824//	-
MSTRG.1103	115349105	115352927	ncbi_1001539	--	3691	-	-	-	-	-	-	-
XR_00234669	66691949	66720362	ncbi_1006247	UGT2A3	2263	Metabolism;(	Global and ov	ko01100//Me	K00699;K006	-	-	-
XR_00234669	66691949	66720362	ncbi_1006245	SULT1B1	6684	-	-	-	-	GO:0044424//	GO:0016782//	GO:0006575//
XR_00234646	111737505	112031475	ncbi_1003129	ELOVL6	7194	Metabolism;l	Global and ov	ko01100//Me	K10203;K102	GO:0031227//	GO:0005488//	GO:0001676//
XR_00234686	125755097	125764962	ncbi_1005154	Atoh1	3269	-	-	-	-	GO:0043231//	GO:0000982//	GO:0006357//
XR_00130914	136729195	136754196	ncbi_1005127	PRKG2	501	Organismal S	Sensory syst	ko04740//Olfa	K19477;K194	GO:0005886//	GO:0004690//	GO:0006612//
MSTRG.1242	44574204	44661972	ncbi_397021	TAGLN	6818	-	-	-	-	GO:0044424//	GO:0003779//	GO:0030154//
MSTRG.1246	46891693	46900408	ncbi_397247	NECTIN1	128	Human Disea	Infectious dis	ko05168//Her	K06081;K060	GO:0005911//	GO:0038023//	GO:0000041//
XR_00233568	48272279	48357034	ncbi_1005209	SC5D	80	Metabolism;l	Global and ov	ko01100//Me	K00227;K002	GO:0031224//	GO:0003824//	GO:0006631//
XR_00233617	694335	725378	ncbi_1005112	GLRX2	4444	-	-	-	-	GO:0031981//	GO:0015037//	GO:0000302//
XR_00233636	50174100	50298199	ncbi_1005174	THNSL1	349	-	-	-	-	-	-	-
XR_00233664	6780713	6995892	ncbi_1001566	KATNAL1	2110	-	-	-	-	-	-	-
XR_00233666	15556048	15562600	ncbi_1005118	SLC25A15	4875	-	-	-	-	GO:0019866//	GO:0008324//	GO:0006839//
MSTRG.1430	4955463	4960483	ncbi_1005185	ST6GALNAC	802	-	-	-	-	GO:0031224//	GO:0016757//	GO:0006464//
XR_00233722	39357335	39360855	ncbi_1102560	--	3558	Environment	Signaling mo	ko04060//Cyt	K05408;K054	-	-	-
XR_00233727	44140346	44142843	ncbi_1005112	KSR1	4512	Environment	Signal transd	ko04014//Ras	K14958;K149	-	GO:0004672//	GO:0006464//
MSTRG.1641	135412411	135415883	ncbi_1001258	MUC13	825	-	-	-	-	GO:0005886//	GO:0046983//	GO:0010669//
MSTRG.1675	203188476	203203227	ncbi_1005168	B3galt5	5125	Metabolism;l	Global and ov	ko01100//Me	K03877;K038	GO:0031224//	GO:0016758//	GO:0006464//
MSTRG.1686	6639959	6652323	ncbi_1001528	PIWIL2	7434	Organismal S	Development	ko04320//Dor	K02156	GO:0043186//	GO:0044822//	GO:0006306//
XR_00233889	72350428	72369838	ncbi_1001535	Hk1	6437	Metabolism;(	Global and ov	ko01100//Me	K00844;K008	-	-	-
MSTRG.1775	92360548	92382302	ncbi_1006233	IPMK	1168	Metabolism;l	Global and ov	ko01100//Me	K00915;K009	GO:0043231//	GO:0051766//	GO:0001838//
XR_00233864	120852366	120906266	ncbi_1001571	DUSP5	73	Environment	Signal transd	ko04010//MA	K04459	-	-	-
MSTRG.1860	81937786	81941802	ncbi_1005151	HOXD8	2257	-	-	-	-	GO:0043231//	GO:0001071//	GO:0003002//
XR_00233943	110902838	110906707	ncbi_1005190	FZD5	9277	Human Disea	Cancers;Infe	ko05200//Pat	K02375;K023	-	-	-
MSTRG.1893	132917053	132970281	ncbi_1005224	ALPI	4570	Metabolism;l	Global and ov	ko01100//Me	K01077;K010	GO:0031224//	GO:0016791//	GO:0006796//
MSTRG.1893	132917053	132970281	MSTRG.1893	Pol	1786	Human Disea	Neurodegene	ko05016//Hu	K10408	GO:0005635//	GO:0000166//	GO:0001171//
MSTRG.1974	35856668	35872162	ncbi_1006240	PLAGL2	103	-	-	-	-	GO:0043231//	GO:0000982//	GO:0006357//
XR_135462.4	13998498	14000927	ncbi_1005253	Slc23a2	1566	Organismal S	Digestive sys	ko04977//Vita	K14611	-	-	GO:0044763//
MSTRG.2044	40483796	40491538	ncbi_1006236	NT5C3A	752	Metabolism;l	Global and ov	ko01100//Me	K01081;K010	GO:0043231//	GO:0008252//	GO:0006753//
XR_00234091	25271338	25273767	MSTRG.2137	gag	8637	-	-	-	-	-	-	GO:0006139//
MSTRG.2150	3281	7032	ncbi_1102582	--	3398	-	-	-	-	-	-	-
XR_00234118	110080	134972	ncbi_1102585	BTN1A1	667	-	-	-	-	-	-	-
MSTRG.2166	110080	142432	ncbi_1102585	BTN1A1	667	-	-	-	-	-	-	-
XR_00234118	110084	122647	ncbi_1102585	BTN1A1	671	-	-	-	-	-	-	-
MSTRG.2166	110084	142427	ncbi_1102585	BTN1A1	671	-	-	-	-	-	-	-
MSTRG.2179	129634	148041	ncbi_1005125	BTN1A1	1301	-	-	-	-	-	-	-
XR_00234132	23761	28771	ncbi_1006224	LILRA2	8183	Organismal S	Development	ko04380//Ost	K06512;K065	-	-	-

SI Appendix, Table. S3 Primers used in this study

Name	primers	Tm (°C)	Length (bp)
XR_001302663.2	F: AAAGATGGGAATGCCTGCTCAACC	60	108
	R: CCACTACAGAGCTGGGAAAGAACAC		
MSTRG.16726.3	F: GTGGGAGCAAAGGAGGGAACAAG	60	119
	R: CCATGTGCGGTTGTCGGCTATC		
MSTRG.19894.13	F: GGAGTCCGTGCATGAACCAAGG	60	112
	R: GGATTTCAAGTGGCAGCAGCATTG		
C3	F: TGAGCACAGCCAAAGAAAGGAACC	60	99
	R: TGACTACCAGCAGAGCCAAGAGG		
C4BPA	F: GCCCAGAATGCCAGTATGTGATAGAG	60	141
	R: GGACCAACCAAACCATAGCCAGAG		
GSTO1	F: CGGCATCAAGTCATCAACATCAACC	60	83
	R: AGAACTGGCACCAGACCTGAGG		
18S rRNA	F: CCCACGGAATCGAGAAAGAG	60	132
	R: TTGACGGAAGGGCACCA		
